# Supplementary material for: Investigating the Impact of NMDA Receptor Organization and Biological Sex in the APPswe/PS1dE9 Mouse Model of Alzheimer’s Disease
Source: Int J Mol Sci. 2025 Feb 18;26(4):1737. doi: 10.3390/ijms26041737 (PMC11855313; doi:10.3390/ijms26041737)
Supplement: Supplementary file 1 [file ijms-26-01737-s001.zip › Supplementary figures submitted.pdf]

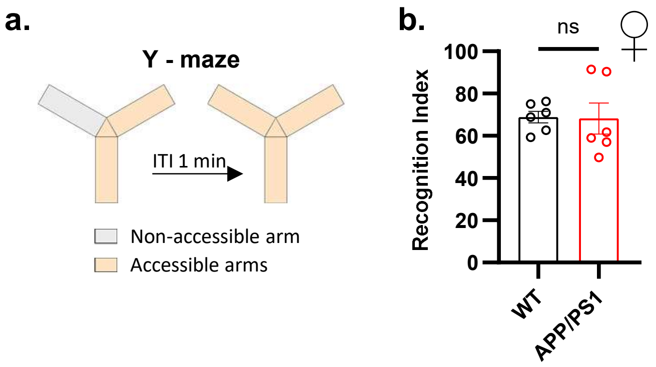

Supplementary Figure S1. Behavioral performance in the Y-maze task with a short inter-trial-interval (ITI). a. Experimental design: Y-maze task, with 1 minute ITI. **b.** Performance of the female APP/PS1 mice was not different from WT when ITI was only 1 min. Data points are individual mice with mean ± SEM. Statistical significance was calculated using the t-test (b). ns corresponds to non-significant.

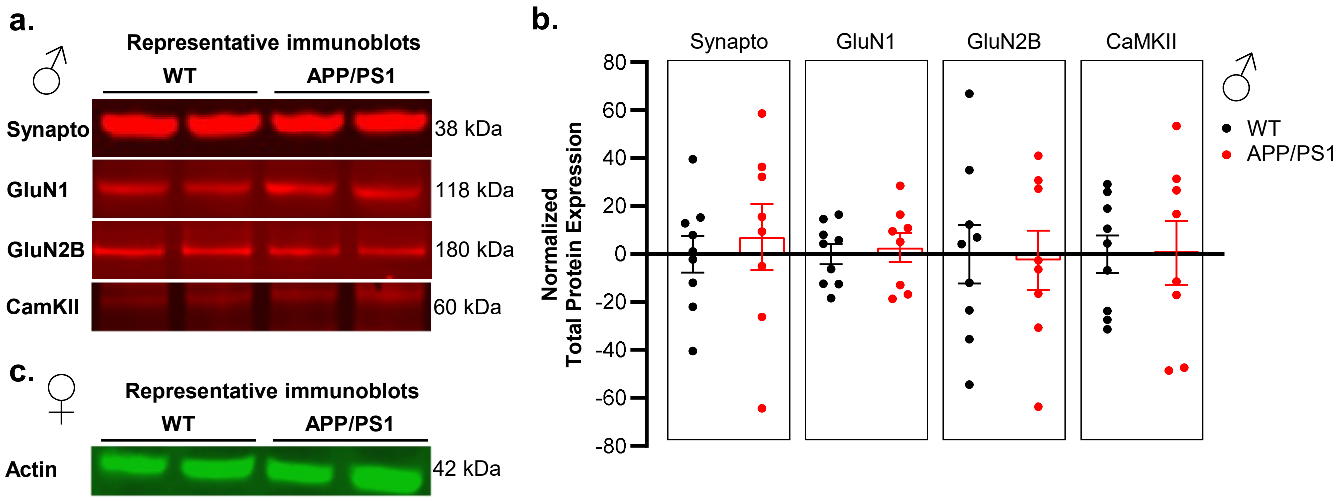

Supplementary Figure S2. Hippocampal expression of cerebral markers in male APP/PS1 and WT. a. Representative Western blots of the different brain markers analyzed in male APP/PS1 and WT mice. **b.** No changes in a level of synatophysin, GluN1 and GluN2B subunits of NMDA receptors, and CaMKII are observed in male APP/PS1 compared to WT. **c.** Representative Western blots of Actin used as a loading control in samples of females APP/PS1 and WT mice. Data points are individual mice with mean ± SEM. Statistical significance was calculated using the t-test.

a.

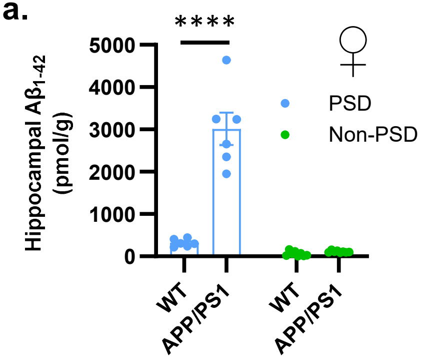

Supplementary Figure S3. Hippocampal accumulation of Aβ1-42 . a. Accumulation of Aβ1-42 is observed exclusively in the hippocampal PSD compartment in female APP/PS1 mice. Data points are individual mice with mean ± SEM. Statistical significance was calculated using two-way ANOVA, ****$P < 0.0001$.

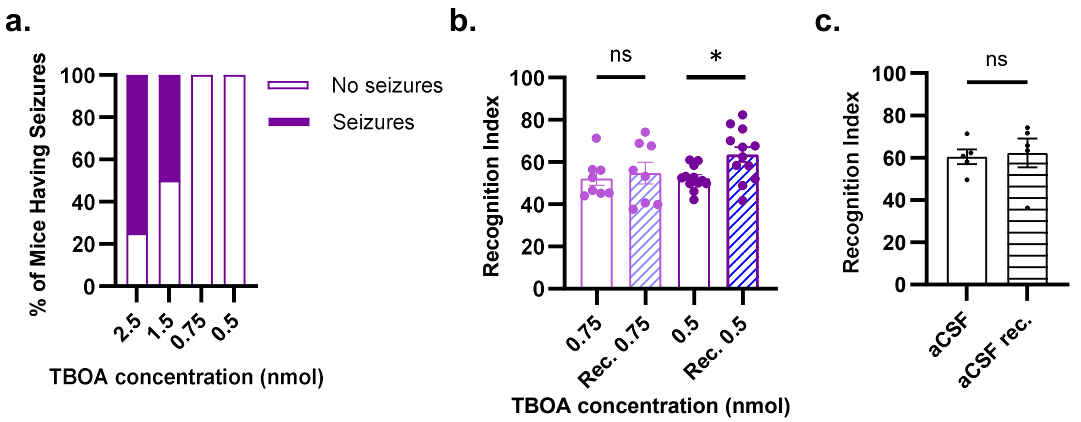

Supplementary Figure S4. DL-TBOA-induced seizures and memory recovery effects in Y-maze performance.
a. DL-TBOA-induced seizures are concentration dependent. **b.** Memory recovery 7-days after the injection of lower DL-TBOA concentrations, that do not provoke seizures, was observed for 0.5nmol but not 0.75nmol DL-TBOA. **c.** Repeated testing in Y-maze with 7 days apart does not affect the performance of the aCSF injected mice. Data points are individual mice with mean ± SEM. Statistical significance was calculated using Paired t-test (b) and Unpaired t-test (c), $^{*}P < 0.05$. ns corresponds to non-significant.
